# A gestational choriocarcinoma of the ovary diagnosed by DNA polymorphic analysis: a case report and systematic review of the literature

**DOI:** 10.1186/s13048-017-0334-3

**Published:** 2017-07-20

**Authors:** Nan Jia, Yan Chen, Xiang Tao, Enzhi Ou, Xin Lu, Weiwei Feng

**Affiliations:** 1 Department of Gynecology, Obstetrics and Gynecology Hospital of Fudan University, 128 Shen Yang Road, Shanghai, 200090 People’s Republic of China; 2 Shanghai Gemple Biotech Co., Ltd, 2F, 3rd Building, Hengyue International Mansion, No.1238 Zhangjiang Road, Pudong New District, Shanghai, 201210 People’s Republic of China; 3 Department of Pathology, Obstetrics and Gynecology Hospital of Fudan University, 128 Shen Yang Road, Shanghai, 200090 People’s Republic of China; 4 Shanghai Key Laboratory of Female Reproductive Endocrine-Related Disease, Fudan University, 413 Zhao Zhou Road, Shanghai, 200011 People’s Republic of China

**Keywords:** Gestational choriocarcinoma of the ovary, DNA polymorphic analysis

## Abstract

**Background:**

Choriocarcinoma of the ovary is rare. This tumor can arise from gestational tissue or pure germ cells of the ovary, the former results in gestational choriocarcinoma. The clinical characteristics and histology of both tumor types are identical, differentiation of these tumors is necessary for effective treatment. One strategy for the differentiation of these tumors types is to identify the presence of paternal DNA by DNA polymorphic analysis.

**Case presentation:**

In the present case, a 27-year-old patient with a history of amenorrhea, lower abdominal pain and vaginal bleeding received a laparoscopic dissection of cystic mass of the right ovary according to an initial diagnosis of ectopic pregnancy. Primary choriocarcinoma of the ovary was diagnosed by pathology, but its origin was uncertain. DNA polymorphic analysis was then performed and a gestational origin was confirmed. The patient subsequently exhibited an excellent response to chemotherapy, achieved complete remission and gave birth to a healthy baby.

**Conclusion:**

Differentiation between two etiologies of primary choriocarcinoma can be achieved with DNA polymorphic analysis and it is necessary to distinguish between them to approach to an appropriate treatment of a patient.

## Background

Primary choriocarcinoma of the ovary can arise from gestational tissue or pure germ cells of the ovary. They are referred to as gestational choriocarcinoma (GCO) or non-gestational choriocarcinoma (NGCO). The estimated incidence of GCO of the ovary is 1:369,000,000 pregnancies, while non-gestational choriocarcinomas correspond to less than 0.6% of ovarian germ cell tumors [1, 2], making this neoplasm very rare. Moreover, both gestational and non-gestational diseases exhibit identical clinical manifestations and histology. The clinical history of pregnancy, amenorrhea, or gestational trophoblastic disease may help to determine the diagnosis, but difficult cases often need DNA analysis, which has not often been performed in the previous reported cases. Saito et al. first described the diagnostic criteria for NGCO in 1963. These include absence of disease in the uterine cavity, pathological confirmation of disease, and exclusion of molar pregnancy and of intrauterine pregnancy [3]. All the criteria were fulfilled in this case, but the presence of paternal DNA revealed the final diagnosis of GCO, indicating that clinical diagnostic criteria are not reliable, except in patients who are unable to conceive or who have never had sexual intercourse [4]. These tumor types should be considered distinct entities with distinct therapeutic approaches, chemotherapy regimens, and prognosis associated with each disease. We summarized 48 cases (ours included) of primary ovarian choriocarcinoma published since 1982. Sixteen more cases reported from 1937 to 1982 are not listed in this article. Although most of the authors declared the reported cases were NGCO, we reanalyzed the information and only 24 NGCO and 2 GCO could be confirmed.

## Case presentation

A 27-year-old married woman (gravida 0) was admitted to a local hospital with a history of 51 days of amenorrhea, lower abdominal pain and vaginal bleeding for 5 days. Her previous menstrual cycles were regular. Her medical history and family history were unremarkable. The general condition of the patient appeared to be good, and pelvic examination revealed a mass in the right adnexal area with tenderness. The urine test showed she was pregnant, and serum β-hCG level was more than 200,000 mIU/ml. Transvaginal ultrasound (TVS) revealed a right adnexal mass and profuse abdominal fluid accumulation.

**Fig. 1:**
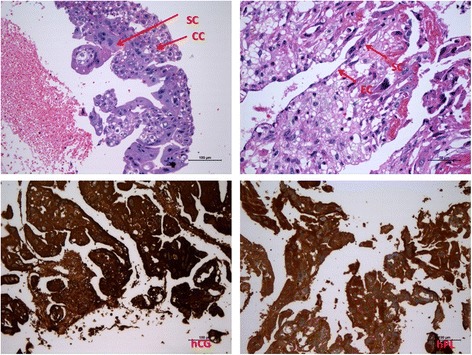
The tumor consists of two types of trophoblastic cells without villus, so choriocarcinoma was diagnosed. SC: syncytiotrophoblastic cells; CC: cytotrophoblastic cells; EC: vascular epithelial cells

According to an initial diagnosis of ectopic pregnancy, laparoscopic exploration was performed. The right ovary was 5*6 cm, partially cystic, ruptured and surrounded by a hematoma. The left ovary and both fallopian tubes were intact. Approximately 500 ml of intraperitoneal blood was noted. The cystic mass of the right ovary was dissected and sent to pathological diagnosis. On the fifth postoperative day, serum β-hCG levels was 14,510 mIU/ml. The patient then transferred to our hospital six days after the surgery. The pathological consult confirmed a pure choriocarcinoma of the right ovary, and an immunohistochemical panel was performed and the samples analyzed were positive for Pan Cytokeratin (AE1/AE3), hCG, human placental lactogen (hPL) and Ki-67(60%), and negative for p53. (Fig. 1).

At the 7th and 10th postoperative day, the serum β-hCG levels fell to 5907 and 2000 mIU/ml, respectively. Further imaging examination was proceeded ten days after the surgery. The contrast pelvic MRI showed the right ovary was 2.1*2.9*3.2 cm, at the front of which a mass of 1.2 cm*1.0 cm was observed. PET-CT showed bilateral ovarian nodules with hypermetabolism, physiological uptake considered, no other specific abnormalities were observed. Other related tests were examined: CA125 (cancer antigen 125): 70.81 U/ml, AFP (alpha fetoprotein): 2.28 ng/ml. As the endometrium thickness was only 5 mm, endometrial biopsy had not been performed.

The patient received five courses of EP-EMA chemotherapy, including cisplatin (80 mg/m2) and etoposide (100 mg/m2), D1; etoposide (100 mg/m2), methotrexate (100 mg/m2 iv and 200 mg/m2 ivgtt), and actinomycin-D (0.5 mg), D7–8, at two-week intervals. Goserelin (3.6 mg) was injected before the beginning of chemotherapy and at four-week intervals during the treatment to protect the ovarian function. During the chemotherapy, the patient was monitored weekly for serum levels of β-hCG, and a rapidly decrease was detected. We observed normalization of the CA125 serum level after one course of chemotherapy. The β-hCG level decreased to normal after two and a half courses of chemotherapy and remained normal thereafter. The contrast pelvic MRIs performed once a month during the chemotherapy showed reduced lesion which became undetectable during the fourth course. The patient remains without evidence of disease 32 months after chemotherapy, her menstruation recovered 12 months after chemotherapy, and gave birth to a healthy baby 25 months after chemotherapy.

Individual DNA polymorphic analysis was used to verify the presence or absence of paternal genetic material. DNA from paraffin-embedded tumor tissue was compared to the patients’ and her husband’s peripheral blood DNA. Manual microdissection of the tumor cells was performed to eliminate the contamination of maternal DNA. Following extraction of DNA from the formalin-fixed and paraffin wax embedded material (QIAamp DNA FFPE Tissue Kit, Qiagen, Valencia, CA, USA), and from blood samples (ZR Genomic DNA-Tissue MiniPrep Kit, Zymo Research, CA, USA) all samples were quantified by NanoDrop (Thermo Scientific, Wilmington, USA), and MicroreaderTM 21 ID system, MicroreaderTM 23sp ID system (Beijing Microread Genetics Co., Ltd., Beijing, China) were respectively used to amplify 10 ng DNA from each biopsy and blood samples. Amplified products were then detected using an ABI 3730xl Genetic Analyzer (Applied Biosystems, CA, USA). Electrophoresis results were analyzed using GeneMapper® ID v.3.2 (Applied Biosystems, CA, USA), and the genetic profiles of the biopsy and peripheral blood were compared.

**Fig. 2:**
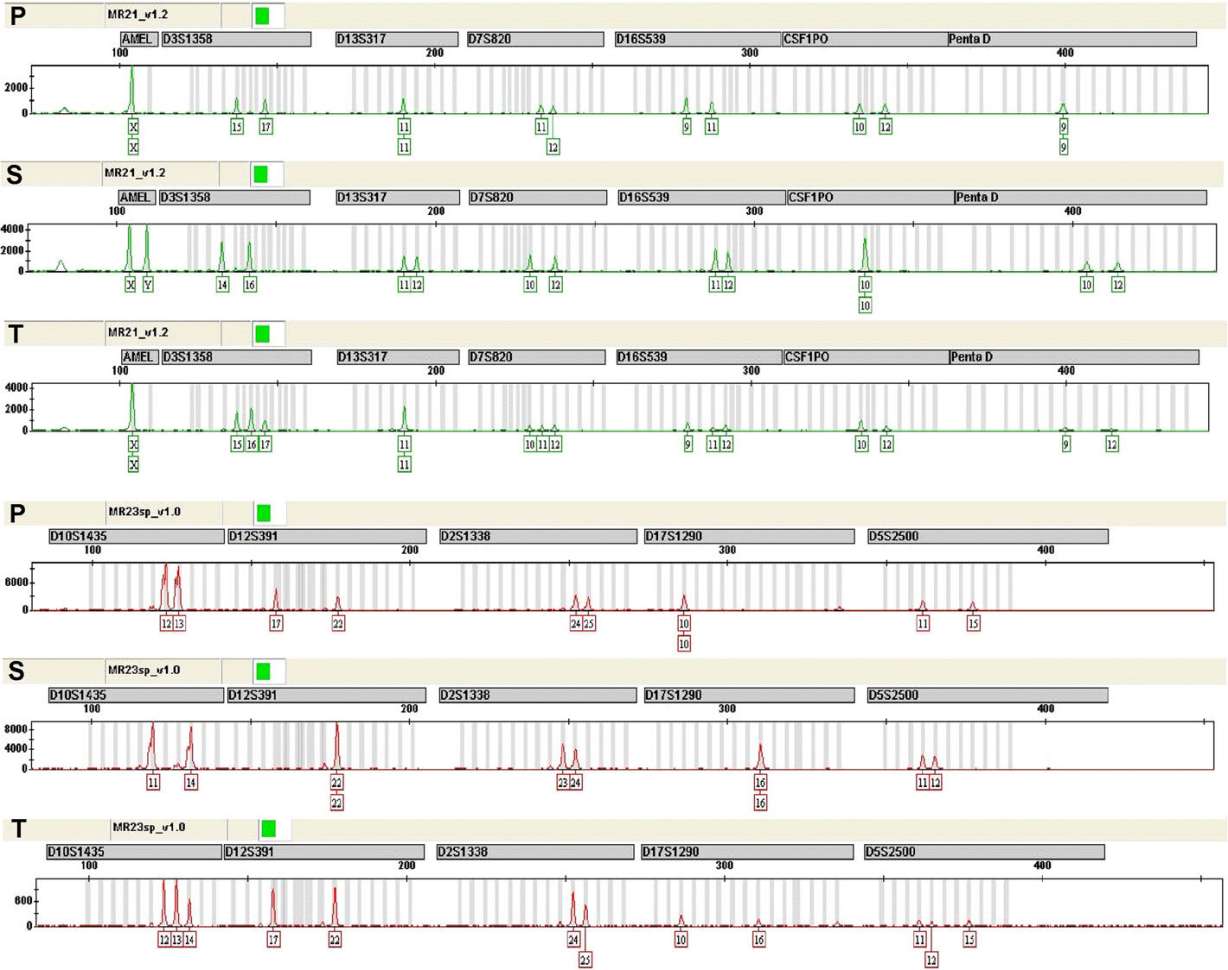
STRs analysis of the case. DNAs from the patient, spouse and tumor were amplified for 43 loci (21 not shown). P, patient; S, spouse; T, tumor

We studied the genetic profiles of 43 highly polymorphic short tandem repeats (STRs) in DNA samples prepared from the patient, spouse and tumor. At 25/43 loci examined, the tumor specimen was shown to contain the paternal allele but not the maternal DNA (D21S11, D18S51, D6S1043, D3S1358, D7S820, D16S539, Penta D, D2S441, vWA, TPOX, TH01, FGA, D18S535, D19S253, D20S470, D22-GATA198B05, D16S539, D8S1132, D4S2366, D13S325, D9S925, D3S3045, D10S1435, D17S1290, D5S2500). At 18/43 loci examined, it could not be determined whether the tumor contained paternal allele because the patient and spouse shared one or two identical alleles (D19S433, D5S818, AMEL, D13S317, CSF1PO, D8S1179, Penta E, D12S391, D2S1338, D6S477, D15S659, D11S2368, D1S1656, D7S3048, D21S1270, D14S608, D12S391, D2S1338). Therefore, none of the loci could be proved to contain maternal allele only. At 20/43 loci examined, the tumor was triploid, which was in accord with the nuclear-heteromorphism of tumor cells. Twelve representative loci from these analyses were summarized in Table 1 and Fig. 2. In more than half (25/43) of the loci studied we were able to demonstrate the presence of paternal DNA in the tumor, indicating a gestational origin for the tumor.

## Discussion and conclusions

We summarized 48 cases of primary ovarian choriocarcinoma published since 1982 in Table 2 (ours included). Although most of the authors declared the reported cases were non-gestational choriocarcinoma, we reanalyzed the information and only 24 non-gestational choriocarcinoma and 2 gestational choriocarcinoma could be confirmed. The origin of other 22 cases was uncertain.

**Table 1:** Summary of polymorphic loci examined and the allelotypes of patient, spouse and tumor of 12 STRs (21 not shown)

Locus	Alleles						
	Patient		Spouse		Tumor		
Paternal allele contained							
D3S1358	15	17	14	16	15	16	17
D7S820	11	12	10	12	10	11	12
D16S539	9	11	11	12	9	11	12
Penta D	9	9	10	12	9	12	--
D10S1435	12	13	11	14	12	13	14
D17S1290	10	10	16	16	10	16	--
D5S2500	11	15	11	12	11	12	15
Paternal allele not determined							
AMEL	X	X	X	Y	X	X	--
D13S317	11	11	11	12	11	11	--
CSF1PO	10	12	10	10	10	12	--
D12S391	17	22	22	22	17	22	--
D2S1338	24	25	23	24	24	25	--

Of 26 cases with confirmed origin, 19 were diagnosed with NGCO because they were young women with no intercourse [1, 4–18], one was diagnosed with NGCO because of XY gonadal dysgenesis (Swyer syndrome) [19], four were confirmed non-gestational [20–23] and two gestational [24] by DNA analysis. Of patients assigned uncertain etiology, one was deduced GCO because of the presence of a corpus luteum [25], which can be suggestive, but not pathognomonic of gestational etiology; three patients were diagnosed with NGCO because of no intercourse in 10 years (G5P3) [26], long duration from the antecedent pregnancy(G1P1) [2], or husband’s undergoing vasectomy(G4P2) [27]. None of them can be excluded from gestational etiology since GCO has been reported to arise many years after an abortion or molar pregnancy, even in postmenopausal woman [28–31]. Other cases were diagnosed with NGCO simply according to pathology.

How to define the origin of a primary choriocarcinoma of the ovary is difficult by clinical characteristics or traditional methods. The etiology of choriocarcinoma has been ascribed to four different sources: from maternal germ cell; from an ovarian pregnancy; from metastases from a regressed or occult uterine primary; or, in infants, from metastases of the placenta [32]. Choriocarcinoma of the ovary can arise from gestational tissue or pure germ cells of the ovary, and it would be useful to discriminate between tumors of different origins because of distinct therapeutic approaches, chemotherapy regimens, and prognosis [33]. Unfortunately, it is extremely difficult. Both gestational and non-gestational diseases exhibit identical clinical manifestations and histology. Histologically, combining with other germ cell elements such as embryonal carcinoma or dysgerminoma in the tumor imply a non-gestational etiology. When sole choriocarcinoma is present, it is difficult to distinguish the etiology by routine histologic examination, even no significant ultrastructural differences are displayed between non-gestational and gestational choriocarcinoma [5]. HCG level does not distinguish between two types of tumor. The absence of primary lesion in the uterus and the presence of a proliferative endometrium do not imply a primary choriocarcinoma either.

The clinical histology is helpful in assigning the etiology. A patient who is sexually immature, unable to conceive, or who has not engaged in sexually intercourse, must have NGCO. Postpubertal women who have been sexually active or have ever been pregnant, gestational origin is a strong possibility. However, they are assigned uncertain etiology unless the presence of paternal DNA in the tumor was determined. It is considered a non-gestational choriocarcinoma rather than a gestational one with an interval of 15 years or longer between the previous pregnancy and the presentation of choriocarcinoma [2], but this is still controversial.

Molecular diagnostic method has been described long time ago that paternal HLA antigens have been identified in GCO [34]. Short tandem repeats (STRs) are general existed DNA polymorphic loci in human genome, which are of highly specificity, genetic and somatic stability. It is very helpful in diagnosing ovarian choriocarcinoma by detecting paternal alleles of the tumor using STRs analysis. Lorigan was the first reported to diagnose choriocarcinoma by analyze DNA polymorphism [24]. More developed and automated techniques are utilized nowadays and become the golden standard of diagnosis of choriocarcinoma. With the increase of polymorphic loci involved in this analysis (43 loci in this report), a higher accuracy of diagnosis as GCO is concluded for the present case.

Treatment of primary ovary choriocarcinoma should be carefully chosen according to the situation of the patients. In a woman who desires further child-bearing, conservative surgery may be employed if the tumor does not involve the uterus or the other ovary. One patient was pregnant one year after the completion of chemotherapy, and gave birth to a healthy baby [34], and our patient also had the same good outcome. If the tumor is extensive, especially if the etiology is non-gestational, intensive cytoreductive surgery should be performed. Most of the patients under 30 years old (23/34) received conservative surgery, seven underwent radical surgery of total abdominal hysterectomy and salpingo-oophorectomy with or without pelvic lymph node dissection. In our case, the patient’s β-hCG level decreased rapidly after the cystectomy, and became negative during chemotherapy, no lesion was seen in MRI or ultrasound, so we didn’t perform any further surgery.

Advances in chemotherapy significantly promote the survival rate of ovarian choriocarcinoma, and make determinations of the etiology of an ovarian choriocarcinoma important. It is generally accepted that GCO can be treated with methotrexate, actinomycin D or etoposide as a single agent, or with combined agents such as EMA-CO (etoposide, methotrexate, actinomycin D, cyclophosphamide, vincristine) when high risk factors are present. However, NGCO are generally treated with BEP (bleomycin, etoposide, cisplatin) regimen. We assigned an EP-EMA regimen to our patient before the DNA analysis results came out hoping to cover both trophoblastic and germ cell tumor, and received satisfactory results.

It is generally believed that non-gestational choriocarcinoma has a worse outcome than a gestational one. We did not find any differences in prognosis between these two types of tumor probably because of the inadequacy of cases. Most of the patients (20/25) who underwent conservative surgery remained no evidence of disease for 1–16 years. Considering the early onset of non-gestational choriocarcinoma and the sensitivity to chemotherapy of gestational one, we recommend all patients who desire for future pregnancy can receive conservative surgery as long as the contralateral ovary and the uterus are intact.

**Table 2:** Choriocarcinoma of the ovary: a summary of cases

Authors	Age	Reproductive history	β-hCG (mIU/ml)	Surgery	Chemotherapy	Outcome	DNA polymorphic analysis
Gestational choriocacinoma							
Lorigan,1996 [24]	41	NS	151,500	TAH,BSO	BEP then EI	NED	Yes
Our case	27	G0	>200,000	ROC	EP-EMA	NED 32 mo	Yes
Non-gestational choriocacinoma							
Vance,1985 [5]	9	NI	34	RO	VEP	NED 12 mo	No
Raju,1985 [6]	16	NI	NS	Autopsy	None	DOD	No
Axe,1985 [1]	6	NI	Normal	RO	None	NED 10 yrs	No
Axe,1985 [1]	11	NI	Elevated	RO	MAV	DOD	No
Sengupta, 1987 [7]	11	NI	NS	UO	NS	NS	No
Spingler,1990 [19]	20	Swyer syndrome	NS	Yes,NS	NS	DOD	No
Gribbon,1992 [8]	NS	NI	Elevated	Yes,NS	NS	DOD 4 mo	No
Gribbon,1992 [8]	11	NI	Elevated	Yes,NS	NS	NED 1 yr	No
Brown,1993 [9]	11	NI	NS	USO	NS	NED 32 mo	No
Trigueros,1995 [10]	21	NI	200,000	TAH,BSO	PVB	NED 4 yrs	No
Gungor,1999 [11]	16	NI	20,000	TAH, cytodeduction, omentectomy, appendectomy	EMA-CO	DOD 6 mo	No
Inaba,2000 [12]	12	NI	1,100,000	RSO,LOC, conservative debulking surgery	BEP then EIC	NED 12 mo	No
Goswami, 2001 [13]	18	NI	88,385	LSO,ROC,omental and peritoneal biopsies	MA and oral chlorambucil	NED 5 mo	No
Ozdemir, 2002 [14]	13	NI	91,028	RSO	MAC	NED 9 mo	No
Tsujioka, 2003 [20]	19	NS	110,000	LSO	EMA-CO	NS	Yes
Koo,2006 [21]	33	G0	185,000	TAH,BSO,PLND	MAC	NED 18 mo	Yes
Yamamoto,2007 [22]	19	NS	206,949	LO	EMA,then EA	NED 12 mo	Yes
Kong,2009 [4]	10	NI	NS	LSO,partial omentectomy	PVB	NED 3 mo	No
Exman,2013 [23]	24	G1P0	675,713	TAH,BSO	BEP	NED	Yes
Heo,2014 [15]	12	NI	20,257	LSO	BEP	NED 14 mo	No
Hayashi,2015 [16]	10	NI	6600	RSO	BEP	NED 62 mo	No
Xin,2015 [17]	23	NI	18,000	tumor enucleation, LSO, pelvic peritonectomy, PaLND, omentectomy	BEP	NED 9 mo	No
Wang,2016 [18]	13	NI	Elevated	TAH,LSO,debulking surgery	PVB	DOD 3 mo	No
Wang,2016 [18]	13	NI	NS	LSO	NS	Lost follow-up in 1 yr	No
Uncertain etiology							
Jacobs,1982 [33]	30	G1P1	350,000	lumpectomy	MTX	NED 2 yrs	No
Axe,1985 [1]	21	G1P1	Elevated	RO	MAV	NED 8 yrs	No
Axe,1985 [1]	20	NS	Elevated	RO,appendectomy	MTX	NED 16 yrs	No
Axe,1985 [1]	36	G4P2	Elevated	TAH,BSO,appendectomy	MTX	NED 19 yrs	No
Axe,1985 [1]	35	G4P4	Elevated	TAH,BSO,PLND	PBC	NED 9 mo	No
Kim,1990 [35]	16	G0	565,000	TAH,BSO	MAC	DOD during chemo	unknown
Shin,1994 [36]	45	G6P3	132,005	TAH,BSO	MAC	NED 1 yr	unknown
Byeun,1995 [37]	28	G3P2	13,378	RSO	EMA	NED 1 yr	unknown
Balat,2004 [38]	28	NS	13,378	TAH,BSO,PLND	BEP	DOD during chemo	No
Bazot,2004 [39]	38	P0	2,460,000	TAH,BSO	NS	NED 7 yrs	No
Gerson,2005 [25]	33	G5P3	564,000	RSO,TAH,LSO,splenectomy	EMA-CO	NED 12 mo	No
Corakci,2005 [34]	22	G1P1	15,050	TAH,BSO,PLND,PaLND	BEP	NED 25 mo	No
Hirabayashi,2006 [40]	50	G0	704	TAH,RSO,PLND	P(ip) + TC,EP-EMA,P-EMA(ip)	DOD 10 mo	No
Roghaei,2007[41]	47	G5P5, menopause	970	TAH,BSO,PLND	EMA-CO	NED	No
Park,2009 [26]	55	G5P3	64,838	TAH,BSO	BEP	NED 20 mo	No
Mood,2009 [42]	31	G9P1	>1000	RSO	EMA-CE	NED 7 yrs	No
Mood,2009 [42]	32	G3P2	5500	TAH, BSO, debulking surgery and omentectomy	BEP to EMA-CE	NED 5 yrs	No
Gon,2010 [43]	21	G0	279,000	RSO	NS	NS	No
Lv,2011 [2]	48	G1P1	7664	sub-extensive TAH,BSO,PLND,PaLND	BEP	NED 12 mo	No
Choi,2013 [27]	33	G4P2	74,612	LO,ROC	EMA	NED 5 yrs	No
Haruma,2015 [44]	19	G0	373,170	LSO	EMA-CO	NED 10 mo	No
Rao,2015 [45]	26	G1P1	8160	RSO,partial omentectomy, partial splenectomy and right adrenalectomy	BEO	brain metastasis 2 years after primary treatment	No

*NS* not stated, *NI* no intercourse, *NED* no evidence of disease, *DOD* dead of disease, *mo*, months, *yr.* year

In conclusion, Ovarian choriocarcinoma is very rare and aggressive. However, it has the potential to be cured by surgery followed by chemotherapy. Differentiation between two etiologies of the tumor can be achieved with DNA polymorphic analysis to detect the presence of paternal DNA, and it is necessary to distinguish between them to approach to an appropriate treatment, and better prognosis of a patient. Conservative surgery should be first considered in nonparous women, and distinguished regimens of chemotherapy are recommended in different etiology of the tumor. The protection of the ovarian function from the chemotherapy should be highly valued for young patients especially for who desire future pregnancy.

## Abbreviations

GCO: Gestational choriocarcinomas
NGCO: Non-gestational choriocarcinoma
STRs: Short tandem repeats
β-hCG: β-human chorionic gonadotropin

## Surgery

L/ROC: Left/right ovarian cystectomy
L/RO: Left/right oophorectomy
UO: Unilateral oophorectomy
L/RSO: Left/right salpingo-oophorectomy
USO: Unilateral salpingo-oophorectomy
BSO: Bilateral salpingo-oophorectomy
TAH: Total abdominal hysterectomy
PLND: Pelvic lymph node dissection
PaLND: Paraaortic lymph node dissection

## Chemotherapy

A: Actinomycin-D
B: Bleomycin
C: Cyclophosphamide
E: Etoposide
I: Ifosfamide
M: Methotrexate
O: Vincristine
P: Cisplatin
T: Paclitaxel
V: Vincristine
